# Spatial-temporal patterns of ambient fine particulate matter (PM_2.5_) and black carbon (BC) pollution in Accra

**DOI:** 10.1088/1748-9326/ac074a

**Published:** 2021-06-25

**Authors:** Abosede S Alli, Sierra N Clark, Allison Hughes, James Nimo, Josephine Bedford-Moses, Solomon Baah, Jiayuan Wang, Jose Vallarino, Ernest Agyemang, Benjamin Barratt, Andrew Beddows, Frank Kelly, George Owusu, Jill Baumgartner, Michael Brauer, Majid Ezzati, Samuel Agyei-Mensah, Raphael E Arku

**Affiliations:** 1 Department of Environmental Health Sciences, School of Public Health and Health Sciences, University of Massachusetts, Amherst, MA, United States of America; 2 Department of Epidemiology and Biostatistics, School of Public Health, Imperial College, London, United Kingdom; 3 MRC Center for Environment and Health, Imperial College London, London, United Kingdom; 4 Department of Physics, University of Ghana, Legon, Ghana; 5 Harvard T.H. Chan School of Public Health, Boston, MA, United States of America; 6 Department of Geography and Resource Development, University of Ghana, Legon, Ghana; 7 NIHR HPRU in Environmental Exposures and Health, Imperial College London, London, United Kingdom; 8 Institute for Health and Social Policy, McGill University, Montreal, Canada; 9 Department of Epidemiology, Biostatistics, and Occupational Health, McGill University, Montreal, Canada; 10 School of Population and Public Health, The University of British Columbia, Vancouver, Canada; 11 Institute for Health Metrics and Evaluation, University of Washington, Seattle, United States of America; 12 Regional Institute for Population Studies, University of Ghana, Legon, Ghana

**Keywords:** air pollution, fine particulate matter, black carbon, air quality, Ghana, sub-Saharan Africa

## Abstract

Sub-Saharan Africa (SSA) is rapidly urbanizing, and ambient air pollution has emerged as a major environmental health concern in growing cities. Yet, effective air quality management is hindered by limited data. We deployed robust, low-cost and low-power devices in a large-scale measurement campaign and characterized within-city variations in fine particulate matter (PM_2.5_) and black carbon (BC) pollution in Accra, Ghana. Between April 2019 and June 2020, we measured weekly gravimetric (filter-based) and minute-by-minute PM_2.5_ concentrations at 146 unique locations, comprising of 10 fixed (∼1 year) and 136 rotating (7 day) sites covering a range of land-use and source influences. Filters were weighed for mass, and light absorbance (10^−5^m^−1^) of the filters was used as proxy for BC concentration. Year-long data at four fixed sites that were monitored in a previous study (2006–2007) were compared to assess changes in PM_2.5_ concentrations. The mean annual PM_2.5_ across the fixed sites ranged from 26 *μ*g m^−3^ at a peri-urban site to 43 *μ*g m^−3^ at a commercial, business, and industrial (CBI) site. CBI areas had the highest PM_2.5_ levels (mean: 37 *μ*g m^−3^), followed by high-density residential neighborhoods (mean: 36 *μ*g m^−3^), while peri-urban areas recorded the lowest (mean: 26 *μ*g m^−3^). Both PM_2.5_ and BC levels were highest during the dry dusty Harmattan period (mean PM_2.5_: 89 *μ*g m^−3^) compared to non-Harmattan season (mean PM_2.5_: 23 *μ*g m^−3^). PM_2.5_ at all sites peaked at dawn and dusk, coinciding with morning and evening heavy traffic. We found about a 50% reduction (71 vs 37 *μ*g m^−3^) in mean annual PM_2.5_ concentrations when compared to measurements in 2006–2007 in Accra. Ambient PM_2.5_ concentrations in Accra may have plateaued at levels lower than those seen in large Asian megacities. However, levels are still 2- to 4-fold higher than the WHO guideline. Effective and equitable policies are needed to reduce pollution levels and protect public health.

## 1. Introduction

Global PM_2.5_ exposures are gradually declining, but there is little data from sub-Saharan Africa (SSA), where there are increasing concerns about air pollution in cities [1]. The urban population in SSA has increased by over 400% since 1980 to about 450 million people in 2017, making it the world’s fastest urbanizing region [2]. Urban residents in SSA have access to increasing infrastructure, technology, and services for improved quality of life [3, 4]. However, the sprawl has been largely unplanned in terms of land use factors. Environmental protection policies have also not kept pace with urban growth [3, 5], making air quality a growing public health concern in cities [6–8]. Yet, cities in SSA lack ground-level air quality monitoring as exists in North America, Europe, and parts of Asia [9, 10]. This lack of systematic monitoring is an obstacle to understanding the within-city patterns, sources and health impacts of air pollution, which are essential for designing effective air quality policies [7, 11, 12].

Exposure to elevated levels of fine particulate matter (PM_2.5_) and black carbon (BC), a component of PM, presents economic and health risks to urban residents in SSA and elsewhere [13–15]. Evidence suggests that BC is associated with higher health effects per unit when compared to PM mass, and is an indicator of the health risks related to emissions from combustion sources [16]. As SSA urbanizes, there is an urgent need for detailed air monitoring data in cities to inform interventions to protect the health and wellbeing of the population. In particular, city-wide data on BC in SSA cities are limited [14, 17, 18].

In Accra, Ghana’s largest city and capital, air pollution emissions are characterized by diverse mixture of combustion and non-combustion sources, including biomass fuels, road dust and vehicle emissions [17, 19, 20]. Like other cities in SSA, rapid urbanization in Accra is intensifying industrial and economic activities as well as increasing the demand for transportation, new fleet of vehicles, and energy, all with major implications for air quality, exposure patterns and health inequalities [21, 22].

We aimed to collect detailed spatial and temporal data and characterize within-city variations in PM_2.5_ and BC in the Greater Accra Metropolitan Area (GAMA) of Ghana. In a large-scale measurement campaign, we collected year-long data on PM_2.5_ and markers of BC from a network of diverse motoring sites. The data and analysis provide comprehensive and granular information on air pollution variations in a sprawling SSA city. We also analyzed changes in PM_2.5_ concentrations over a decade by comparing annual data with those in a previous smaller study (2006–2007) [23].

## 2. Methods

### 2.1. Study location

The GAMA is the industrial and administrative center of Ghana and one of the fastest growing metropolitan areas in SSA with ∼5 million residents and an annual growth rate of 4.2% [24]. The GAMA consists of Accra Metropolitan Area (AMA) at its core, the port city of Tema to the east and 11 other adjoining districts [25, 26]. The GAMA is in a tropical climate zone with high average monthly temperatures and relative humidity (RH) ranging between 25 °C and 33 °C (77–90 °F) and 77%–85%, respectively [25]. The GAMA has two major seasons: the rainy (May–October) period, and the dry period comprising the Harmattan (November–February) characterized by north-easterly trade winds from the Sahara Desert [27].

### 2.2. Study design

This work was conducted within the multi-country and multi-city ‘Pathways to Equitable Healthy Cities’ study (http://equitablehealthycities.org/), which aims to provide scientific evidence on how urban development and policies can be managed to enhance health equity.

As previously described [27], we designed a year-long campaign to examine the spatial (land-use features) and temporal (daily, weekly, monthly and seasonal) variations in ambient PM_2.5_ and BC by sampling at a combination of fixed (∼1 year, *n* = 10 sites) and rotating (7 d, *n* = 136) sites. This design allowed for detailed assessment of both the temporal (using fixed site data) and spatial (using rotating site data) variability of PM_2.5_ and BC over the study area. Further, this design allowed us to optimally use a finite number of monitoring equipment to capture data across the entire geographical extent of the study area. We used a structured form to collect information on land-use features at each monitoring site [27]. The sites were subsequently grouped into four land-use classes: commercial, business, industrial (CBI); high-density residential; medium/low-density residential; or peri-urban (see supplementary text S1 for additional details). We originally planned a 12 month field campaign to collect data at 150 sites starting April 2019; however, the fieldwork was suspended for six weeks (31st March–18th May 2020) due to the COVID-19 pandemic lockdown in Accra and self-isolation of field team members. After the lockdown was lifted and daily activities returned to pre-lockdown status, we conducted additional three weeks of measurement (19th May–11th June 2020) at all fixed sites along with 12 rotating sites, resulting in close to 12 months of data from 10 fixed and 136 rotating sites (see figure S2 (available online at stacks.iop.org/ERL/16/074013/mmedia) for measurement timeline).

The 10 fixed sites were operated continuously, collecting weekly and 1 min averages throughout the measurement campaign at key locations selected based on population density, road networks, neighborhood socioeconomic status (SES) and household biomass fuel use data from the national census [28]. To compare changes in annual mean PM_2.5_ levels within the last decade, four of the 10 fixed sites were placed at the exact locations monitored by Dionisio and colleagues [23]. We also collected 1-week samples at each of the 136 rotating sites, which were selected with a stratified random sampling scheme based on land-use, with more emphasis placed on AMA where the majority of the population live [27]. The sites were initially computer-generated and the actual sampling locations that were as close as possible to the computer-generated ones were identified by the field team. The median distance (interquartile range, IQR) between the original computer-generated locations versus the actual sites monitored was 181 (67–407) m. During the field campaign, the rotating sites were sampled in groups of five each measurement week alongside the fixed sites.

### 2.3. PM_2.5_ measurement and analytical methods

We measured both real-time (1 min interval) and integrated gravimetric (weekly averages) PM_2.5_ concentrations using portable battery operated low-cost and low-power monitors that were placed in protective cases fastened on metal poles at about 4 m (±1 m) above ground [27]. We included in our analysis only samples from monitors that operated for ⩾75% of the measurement period (i.e. at least 5 out of 7 d to capture both weekdays and weekends) and had an average flow rate within 10% of the intended rate.

### 2.4. Integrated PM_2.5_

Weekly integrated PM_2.5_ was measured using the Ultrasonic Personal Aerosol Sampler (UPAS) (Access Sensor Technologies, Fort Collins, USA) [29] operated at 1 litre per minute (lpm). The UPAS has been demonstrated to have a close agreement with reference monitors [29–31] over a wide range of concentrations (10–1600 *μ*g m^−3^) in diverse settings. However, a recent field evaluation suggested that overloading could occur at filter masses above 650 *μ*g [32], an issue that could be avoided by using the duty-cycle feature on the UPAS in highly polluted environments. To avoid overloading filters and to also conserve battery power, the UPAS was operated at 50% duty cycle, drawing air 30 s every minute for a total of 5040 min over the 7 d sampling period. PM_2.5_ mass was collected on 2 *µ*m pore size 37 mm barcoded Teflon membrane filters (https://mtlcorp.com/filters/) and weighed pre- and post-sampling using a MTL AH500 automated robotic scale (www.mtlcorp.com/#/filter-weighing) maintained in a temperature and RH controlled laboratory (23 ± 2 °C, 35 ± 2% RH) at The University of British Columbia. Further information on the UPAS and filter handling can be found elsewhere [27, 33]. An additional 27 duplicate (20% of sites) integrated samples and 28 field blanks were collected at rotating sites, including three post-COVID-19 lockdown duplicates and blanks. The average of the duplicate measurements was taken and final PM_2.5_ concentrations were blank corrected. Quantitative information on blanks and duplicates are in the supplementary text (figure S3).

### 2.5. Continuous PM_2.5_

We deployed a low-cost Zefan real-time continuous monitor (www.zfznkj.com/) to measure PM_2.5_ concentrations at 1 min intervals. The Zefan relies on a light scattering technique to assess PM_2.5_ using Plantower sensors (model PMS7003), which have been evaluated with reference monitors (i.e. FDMS 8500 and TEOM 1400ab) over 6–12 month periods [34, 35]. While this technique provides accurate temporal pattern in measured PM concentrations, its magnitude is inexact as PM mass are only inferred from particle characteristics (e.g. number, size and refractive index), which can be affected by weather conditions (e.g. RH and temperature) [34, 36].

Following previous studies [23, 37], we corrected the minute-by-minute continuous PM measurements by a correction factor (CF) calculated such that the average of continuous PM_2.5_ measurements was equal to the integrated gravimetric PM_2.5_ concentration at the same location over the same 7 d measurement period. This was done to ensure that the average weekly continuous measurements were the same as the gravimetric which has less error than optical sensors. We calculated unique CFs per site for each 7 d period. The median (IQR) of the CFs were 0.84 (0.69–1.13), similar to CFs previously reported for a different optical sensor in Accra [23, 37].

We tested minute-by-minute monitor-to-monitor precision by running all monitors alongside each other over a 24 h period prior to the commencement of field campaign [27]. Further, we conducted mid-campaign (in January 2020) monitor-monitor precision by co-locating the instruments at one of the fixed sites for a week to assess potential drift over the course of the campaign. Finally, post-campaign, we co-located two Zefan sensors with a U.S. federal reference monitor located at the U.S. embassy in Accra. We did not see any within- or between-monitor bias in the sensor performance pre-, mid-, and post-campaign.

### 2.6. Black carbon

Black carbon (BC) aerosols are known indicators of combustion-related constituents of PM emissions and contribute to global warming [38, 39]. Recent epidemiological studies also indicate associations between BC and adverse health outcomes [16, 40]. Thus, we used the absorption coefficient (light absorbance) (10^−5^m^−1^) of the post-weighed PM_2.5_ filters, estimated by applying an image-based reflectance method [41], as a marker for BC concentrations [42, 43]. The image-based reflectance method closely correlates (*r* ^2^ = 0.98) to elemental carbon (EC) concentrations by thermo-optical reflectance, with 1 absorbance unit (1 × 10^−5^m^−1^) equivalent to 1.67 *µ*gm^−3^ EC [41].

## 3. Data analysis

We collected 99 313 h (10 fixed sites = 78 890 and 136 rotating sites = 20 423) of valid real-time and 654 (fixed sites = 518 and rotating sites = 136) weekly integrated gravimetric PM_2.5_ samples. Of these, 21 (fixed) and 10 (rotating) integrated samples were excluded from analysis either due to failure to meet inclusion criteria or for quality control reasons (e.g. blocked airflow and SD card malfunction), leaving a total of 623 weekly (497 fixed and 126 rotating sites) gravimetric samples for analysis.

### 3.1. Spatial analysis

We used data from the rotating sites to assess the spatial patterns of PM_2.5_ and BC across the city by the four site-types: CBI, high-, and medium/low-density residential and peri-urban. To provide more detail on influence of traffic related sources on PM_2.5_ pollution in the GAMA, we grouped the samples collected at rotating sites according to the type (major, secondary and minor) and surface material (paved, mixed and unpaved) of the road near the monitoring site. Since monitoring at rotating sites occurred in groups of five sites per week (i.e. samples were not collected simultaneously at all rotating sites, nor evenly by site-types during each measurement week), we accounted for potential influence of time trend/season on the spatial patterns of the measured concentrations to allow for comparison across sites. We adjusted for potential time trends at the rotating sites by applying weekly specific temporal adjustment factor (TAF) using data from the ten fixed (year-long) sites. For each measurement week, a TAF calculated as the ratio of the mean PM_2.5_ or BC across all fixed sites for that week to the mean annual PM_2.5_ or BC across all fixed sites was used to adjust the samples collected at the rotating sites in that particular week [44]. The season adjusted concentration $({C_i})_{j{ }}^{{\text{adjusted}}}$ of the *i*th rotating site for the *j*th measurement week was calculated as:

\begin{equation*}({C_i})_j^{{\text{adjusted}}} = {({C_i})_j}/\left[ \left( C^{{\text{Fixed Site}}}\right)_j/\left( {\overline {C^{{\text{Fixed Site}}}} } \right) \right]\end{equation*}

where ${({C_i})_j}\,$is the PM_2.5_ or BC concentration measured at the *i*th rotating site in the *j*th measurement week; ${(C^{{\text{Fixed Site}}})_j}$ and $\left( {\overline {C^{{\text{Fixed Site}}}} } \right)$ are the average PM_2.5_ or BC in the corresponding *jth* measurement week and annual average PM_2.5_ or BC at all fixed sites respectively, and $\left[ \left( C^{{\text{Fixed Site}}}\right)_j/\left( {\overline {C^{{\text{Fixed Site}}}} } \right) \right]$ is the TAF.

### 3.2. Temporal analysis

We examined the temporal patterns in the data by season (Harmattan vs non-Harmattan), days of the week (plus weekday vs weekend), and time of day (diurnal) using data from the fixed sites. We also evaluated changes in annual PM_2.5_ levels over a decade (2006–2007 vs 2019–2020) by comparing fixed site data obtained from the same four residential locations sampled in a previous study [23].

All analyses were done using the statistical analysis package R, version 3.6.1 [45], and an alpha of 0.05 was used as cut-off of significance.

## 4. Results

### 4.1. Spatial patterns in PM_2.5_ and BC concentrations

**Figure 1.**
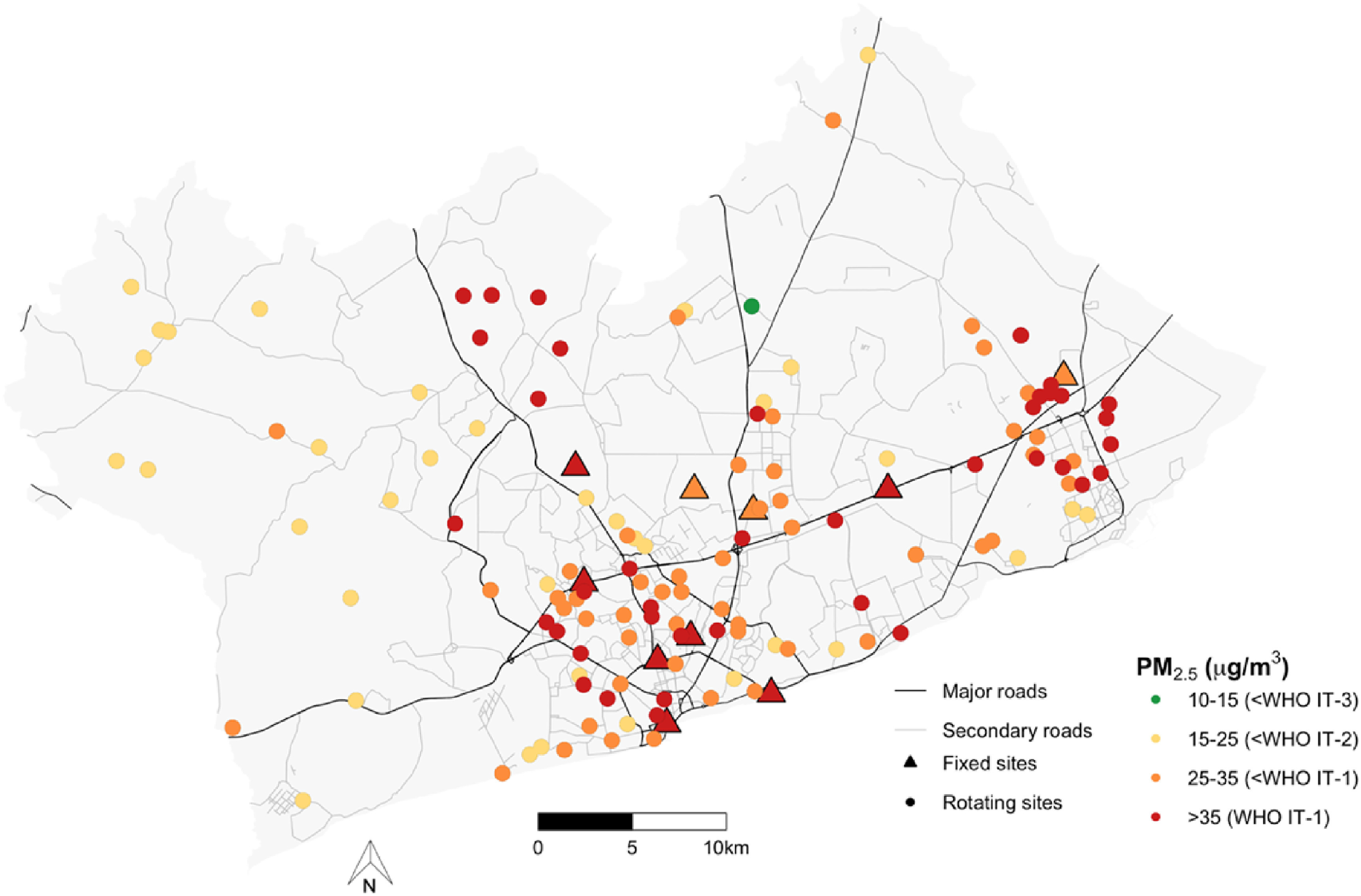
Year-long (fixed) and week-long (rotating) monitoring locations. The colors indicate the integrated PM_2.5_ concentration relative to the World Health Organization (WHO) Air Quality Guidelines (AQG) for PM_2.5_ (IT = interim target). The average concentrations at the fixed sites represent the overall mean of 52 weeks, while the rotating sites represent seasonally-adjusted values (also representing estimated annual means). Major and secondary/tertiary roads are from OpenStreetMap (downloaded 2019) and the GAMA boundary from Ghana Statistical Service.

**Figure 2.**
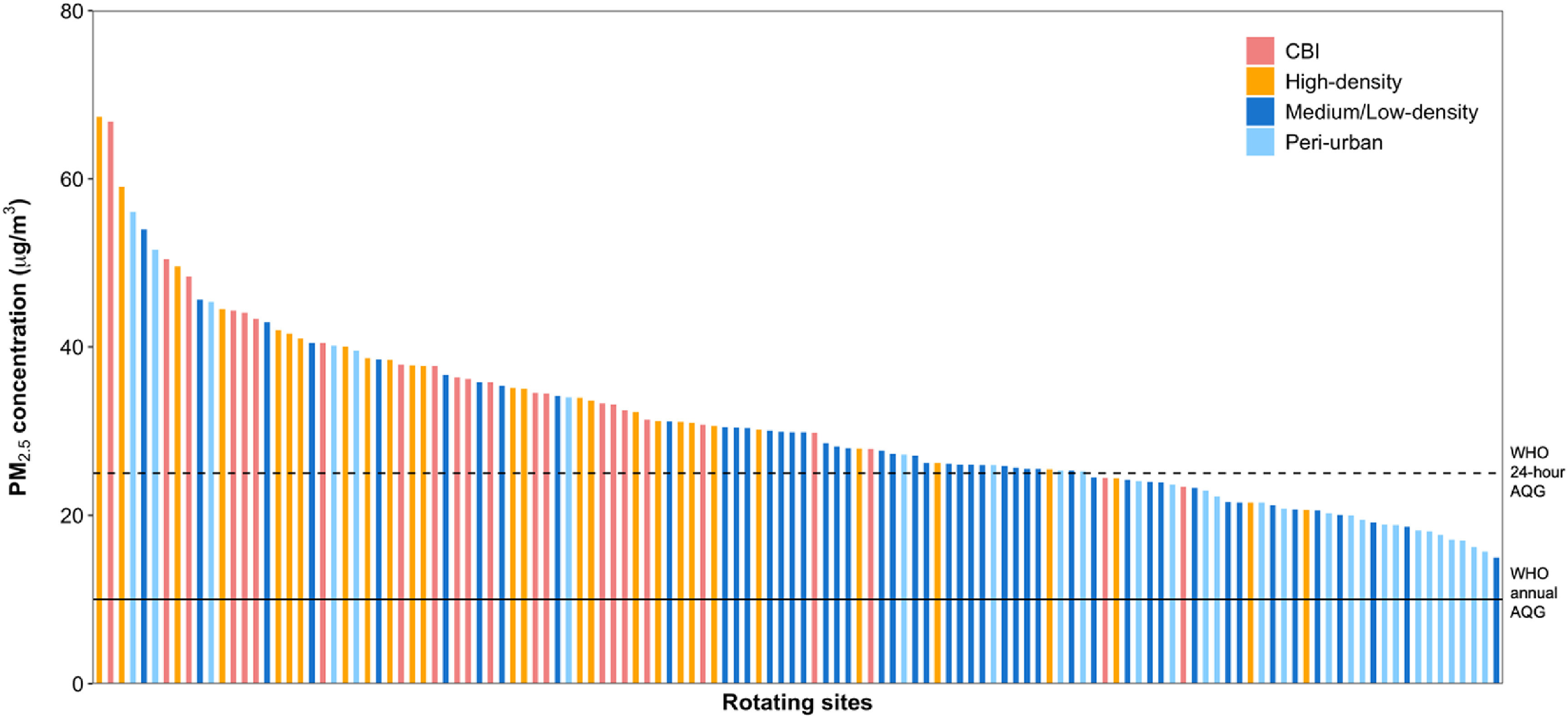
Season-adjusted mean PM_2.5_ concentration at rotating sites by land-use categories. The solid and dashed horizontal lines show WHO annual (10 *μ*g m^−3^) and 24-h (25 *μ*g m^−3^) AQG for PM_2.5_, respectively.

The measurement locations and the measured concentrations relative to the World Health Organization (WHO) air quality guideline are shown in figure 1. The season adjusted mean (standard deviation, SD) integrated PM_2.5_ and BC concentrations across the rotating sites were 31 (10) *μ*g m^−3^ and 5 (2) × 10^−5^m^−1^ respectively. PM_2.5_ concentration at every rotating site was higher than the WHO annual guideline of 10 ug m^−3^, while 99%, 71% and 31% of the sites exceeded the interim target 3 (IT-3, 15 *μ*g m^−3^), IT-2 (25 *μ*g m^−3^) and IT-1 (35 *μ*g m^−3^), respectively (figure 1). The mean PM_2.5_ and BC levels at rotating sites varied by land-use. The highest PM_2.5_ concentrations were in CBI areas (mean: 37; range: 23–67 *μ*g m^−3^) and high-density residential neighborhoods (mean: 36, range: 21–67 *μ*g m^−3^) (*p* < 0.01). Peri-urban sites had the lowest concentrations (mean: 26, range: 16–56 *μ*g m^−3^) after medium/low-density neighborhoods (mean: 28, range: 15–54 *μ*g m^−3^) (figure 2). Similarly, BC concentrations were two times higher in CBI areas (mean: 7, range: 1–14 × 10^−5^m^−1^) compared with peri-urban sites (mean: 3, range: 1–6 × 10^−5^m^−1^) (table 1). In general, average PM_2.5_ concentrations were slightly higher at sites along major and secondary roads compared with sites near minor roads, but not by road surface. We observed similar patterns for BC. Overall, the relative differences in BC across land use factors were much larger than the relative differences in PM_2.5_ concentrations, suggesting that PM_2.5_ in the GAMA may not be affected by community/local sources (such as vehicle tailpipe emissions and trash burning) as much as BC.

**Table 1.** Season-adjusted PM_2.5_ and BC concentrations at rotating sites by land-use categories.

	PM_2.5_ (*µ*g m^−3^)		BC (1 × 10^−5^m^−1^)	
Site type (no. of sites)	Mean (SD)	Range	Mean (SD)	Range
All rotating sites (*n* = 126)	31 (10)	15–67	5 (2)	1–14
CBI (*n* = 23)	37 (10)	23–67	7 (3)	1–14
High-density (*n* = 28)	36 (10)	21–67	6 (2)	2–10
Medium/low-density (*n* = 47)	28 (7)	15–54	4 (1)	1–8
Peri-urban (*n* = 28)	26 (11)	16–56	3 (1)	1–6

## 5. Temporal patterns

### 5.1. Annual and seasonal patterns in PM_2.5_

**Figure 3.**
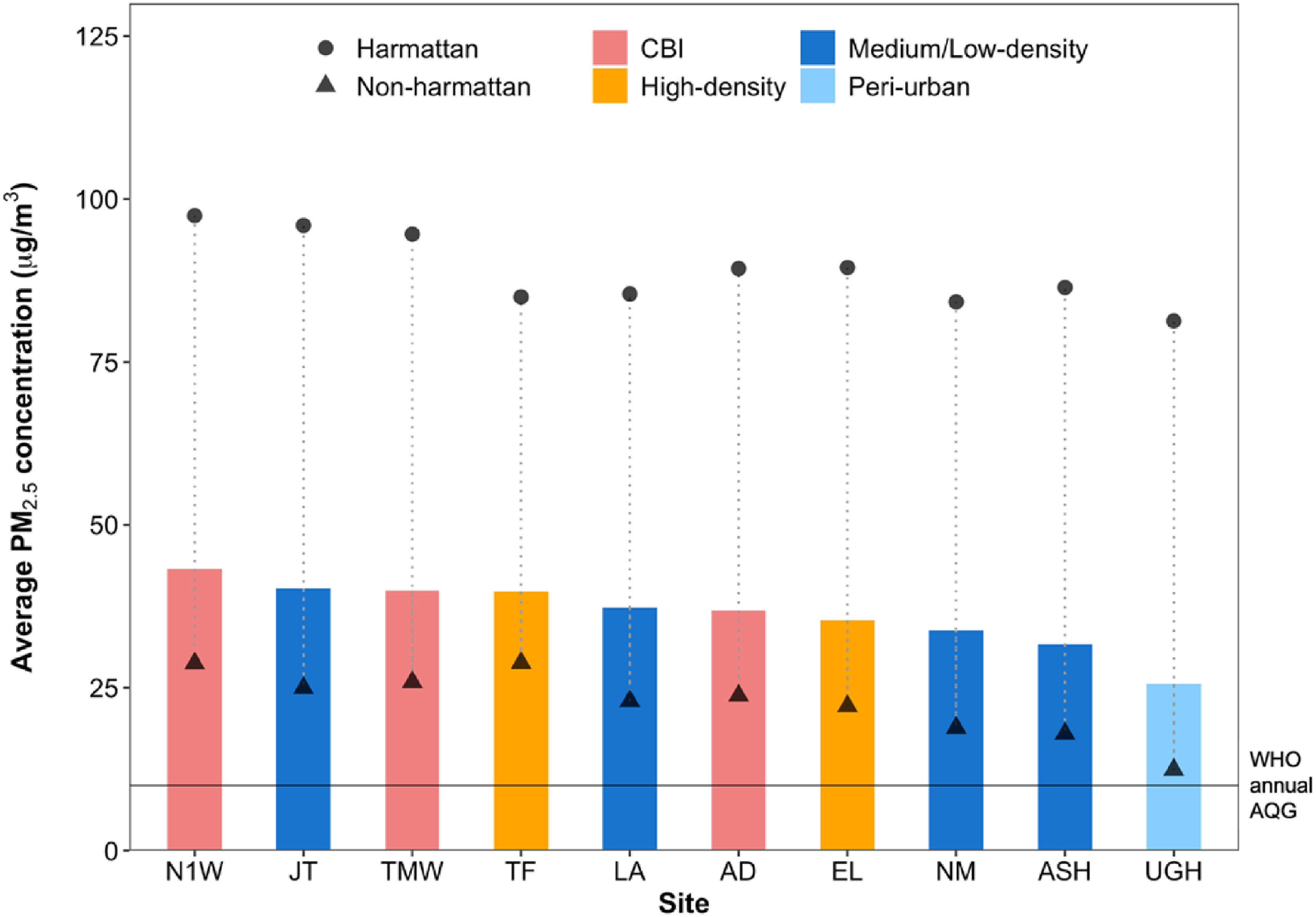
Mean annual PM_2.5_ concentrations (bars; colored by site-type) and mean concentrations by season (Harmattan vs non-Harmattan). The solid horizontal line shows the WHO annual AQG of 10 *μ*g m^−3^. The dotted line represents the magnitude of the difference between seasonal non-Harmattan and Harmattan mean concentrations. CBI: Commercial, business and industrial areas. Sites: N1 West at Lapaz (**N1W**) and Tema Motorway (**TMW**) are at the west and east ends of the multi-lane N1 motorway; Asylum Down (**AD**) is on the Ring Road Central; Jamestown (**JT**) and Nima (**NM**) are low-income, densely populated and high biomass use neighborhoods in south and middle of AMA; Taifa (**TF**) is an emerging neighborhood north of the city; Labadi (**LA**) is an indigenous Ga community along on the Coast; East Legon (**EL**) is a high-income neighborhood next to the University of Ghana Campus. Previously residential streets in EL now host large corporate, commercial and small business ventures; Ashaiman (**ASH**) is an emerging neighborhood next to the port city of Tema; and University of Ghana Hill (**UGH**) is located on top of the quiet Legon Hill.

Mean (SD) annual PM_2.5_ concentrations across the ten year-long (fixed) sites was 37 (40) *μ*g m^−3^ and ranged from site-type specific annual means of 26 *μ*g m^−3^ at the peri-urban site, 32–40 *μ*g m^−3^ at medium/low-density residential sites, 35–40 *μ*g m^−3^ at high-density residential sites, and 37–43 *μ*g m^−3^ at CBI areas (figure 3). Similarly, annual mean BC concentrations were lowest at the peri-urban site and highest at CBI sites (table 2).

**Table 2.** Annual and seasonal PM_2.5_ and BC concentrations at fixed (yearlong) sites by land-use categories.

		PM_2.5_ (*µ*g m^−3^)		BC (1 × 10^−5^m^−1^)	
Site type (no. of sites)	Season	Mean (SD)	Range	Mean (SD)	Range
Fixed sites (*n* = 10)	Annual	37 (40)	6–266	7 (4)	1–25
	Harmattan	89 (64)	24–266	12 (5)	3–25
	Non-Harmattan	23 (7)	6–52	6 (3)	1–18
CBI (*n* = 3)	Annual	40 (41)	17–266	11 (4)	3–25
	Harmattan	94 (67)	28–266	16 (5)	5–25
	Non-Harmattan	26 (5)	17–52	10 (3)	3–17
High-density (*n* = 2)	Annual	38 (37)	16–231	7 (3)	3–21
	Harmattan	87 (63)	26–231	12 (4)	5–21
	Non-Harmattan	26 (6)	16–41	6 (2)	3–12
Medium/low-density (*n* = 4)	Annual	36 (41)	11–245	6 (4)	1–22
	Harmattan	88 (64)	24–245	10 (4)	3–22
	Non-Harmattan	21 (7)	11–51	5 (2)	1–18
Peri-urban (*n* = 1)	Annual	26 (41)	6–217	3 (3)	1–14
	Harmattan	81 (71)	25–217	7 (4)	3–14
	Non-Harmattan	12 (4)	6–26	2 (1)	1–4

**Figure 4.**
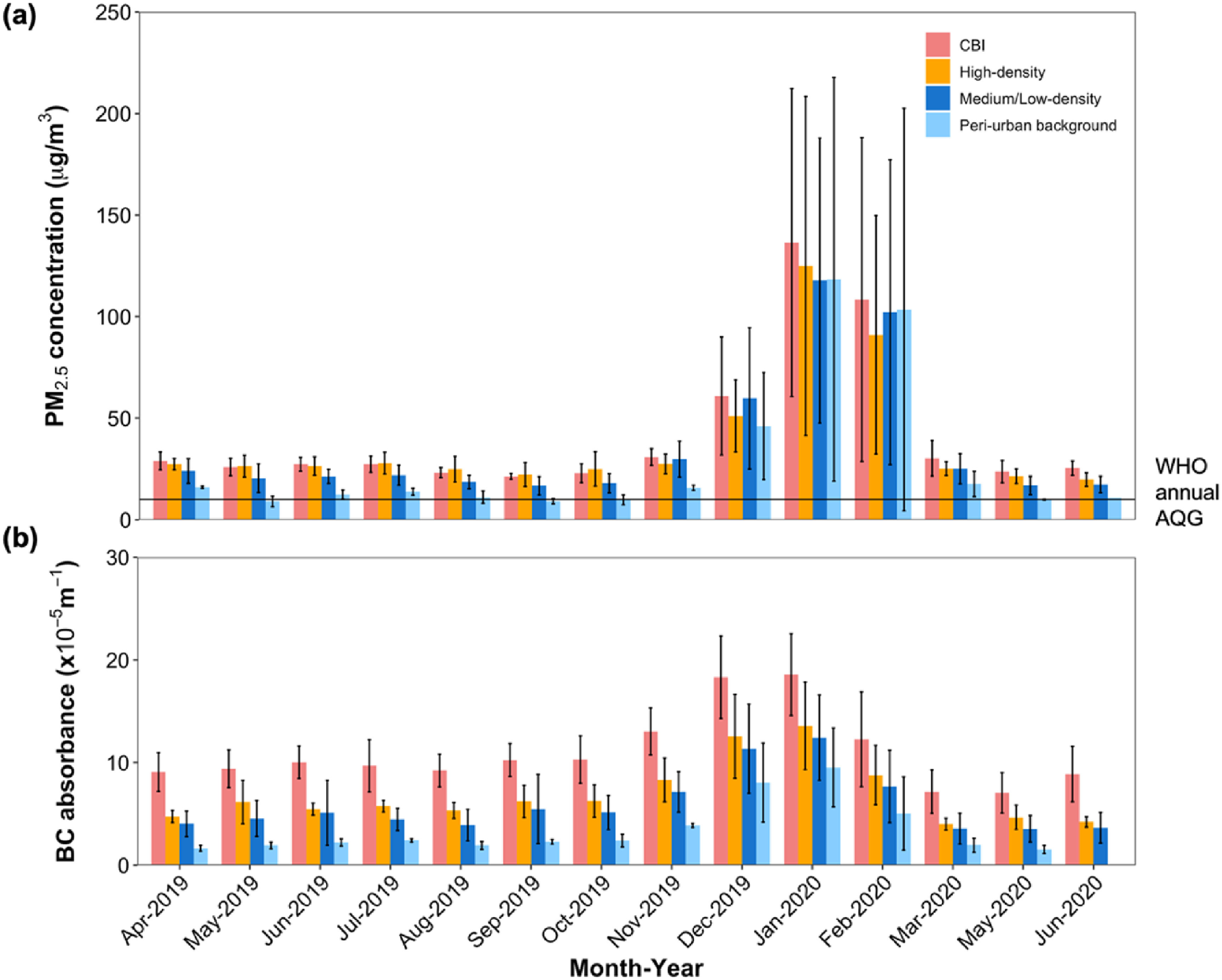
Weekly integrated PM_2.5_ (A) and BC (B) concentrations at the fixed sites averaged by site-types across measurement months. Bars are standard deviations of the weekly measurements in that month. The horizontal line in (A) shows the WHO annual AQG of 10 *μ*g m^−3^.

By season, the mean PM_2.5_ and BC concentrations during the Harmattan (89 *μ*g m^−3^ and 12 × 10^−5^m^−1^) were 4- and 2-fold higher than the non-Harmattan period (23 *μ*g m^−3^ and 6 × 10^−5^m^−1^), respectively (figure 4). The absolute mean difference in PM_2.5_ concentrations between the Harmattan and non-Harmattan periods at each site were between 56 and 71 *μ*g m^−3^ (figure 3). While the absolute levels were higher during the Harmattan, both periods showed substantial relative spatial variability. The peri-urban site recorded the highest seasonal mean difference in PM_2.5_ concentrations while sites in high-density residential neighborhoods recorded the lowest. For each measurement month, the peri-urban site consistently registered the lowest PM_2.5_ and BC levels (figure 4). Like PM_2.5_, BC levels also increased during the Harmattan months (figure 4(b)), and both showed higher variability in the Harmattan as indicated by the sample SD. The overall observed doubling of BC levels in the Harmattan period is noteworthy as it indicates that meteorological conditions likely magnify local emissions and lead to higher concentrations.

### 5.2. Day of the week pattern

Using the minute-by-minute continuous data, we found no differences in mean PM_2.5_ concentrations between day of the week (Monday–Sunday) nor between weekdays and weekends in the GAMA, regardless of whether the data were from the fixed or rotating sites or both (*p* > 0.05). Although Sundays showed slightly lower mean PM_2.5_ overall, the mean difference (3 *μ*g m^−3^) was not significant (*p* = 0.57). The absence of between-day of the week variation in PM_2.5_ in the GAMA was consistent across all land-use categories (figure S4).

### 5.3. Diurnal patterns

**Figure 5.**
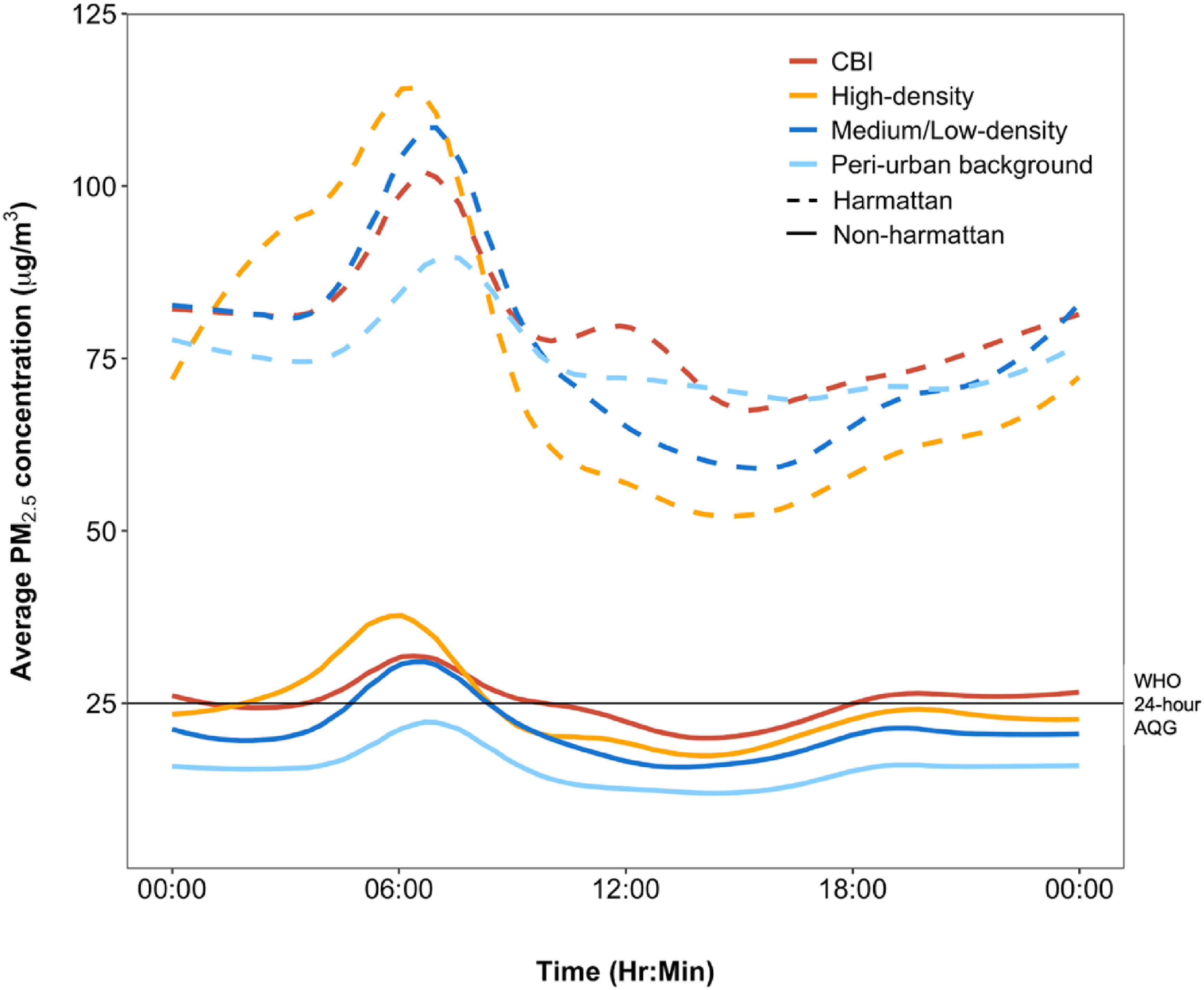
Diurnal patterns of PM_2.5_ concentration across land-use categories. The minute-by minute measurements from all 4592 site-days over the measurement period were averaged. The solid and dashed lines represent Non-Harmattan and Harmattan seasons, respectively. The horizontal line represents the WHO 24 h AQG of 25 *μ*g m^−3^.

PM_2.5_ concentrations from all sites showed strong bimodal variability across time of day, and was consistent over land-use areas and by season (figure 5). PM_2.5_ concentrations at all sites rose around 03:00 daily, peaking at about 06:00, followed by a gradual decline to their lowest values around 10:00. Levels remained fairly stable between 10:00 and 15:00, after which the concentration slowly increased with a relatively smaller peak around 18:00–19:00. There was about an hour delay in the timing of the peaks during the Harmattan and the smaller early evening peak was less pronounced compared to the non-Harmattan period. In general, average PM_2.5_ concentrations at nighttime (18:00–05:59) were slightly higher than daytime levels (37 vs 34 *μ*g m^−3^). During these periods, biomass is burned in some neighborhoods for residential and small-scale commercial purposes, such as cooking street food and bakery operation.

### 5.4. Change in PM_2.5_ concentration since 2006/2007

**Figure 6.**
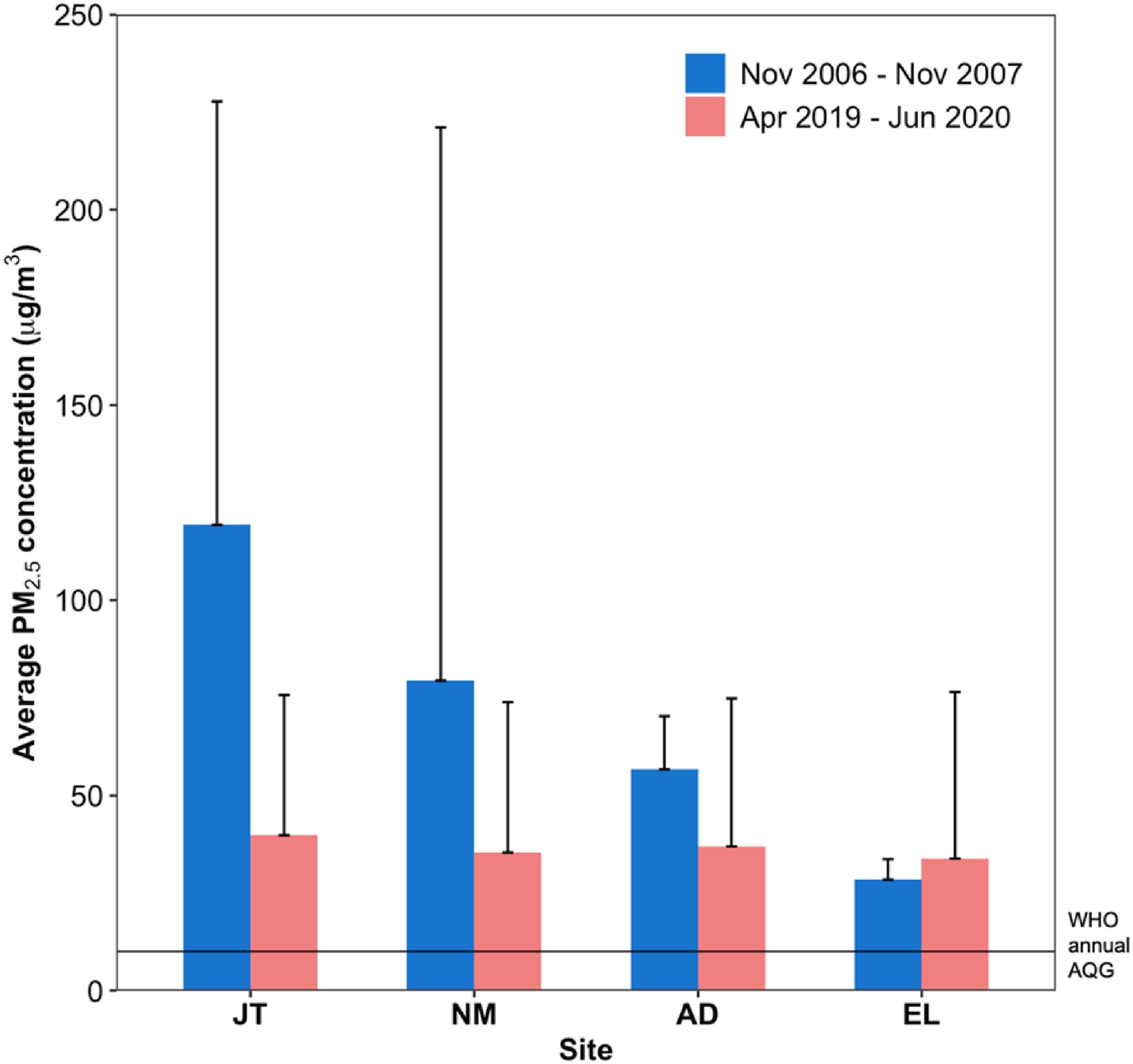
Comparison of mean annual PM_2.5_ concentrations between 2006/2007 (sample range: 12–1292 *μ*g m^−3^) and 2019/2020 (sample range: 13–245 *μ*g m^−3^) measurement campaigns. Bars are standard deviation of all measurements in that study period, including Harmattan. The horizontal line shows the WHO annual AQG (10 *μ*g m^−3^).

In 2006/2007, Dionisio and colleagues [23] recorded large variability (with wide SDs) in mean annual PM_2.5_ in four residential neighborhoods of varying SES and biomass use within the AMA, with values ranging from 28 *μ*g m^−3^ in the affluent neighborhood of East Legon (EL), and 57 *μ*g m^−3^ in middle-income Asylum Down (AD), to >70 *μ*g m^−3^ in low-income, densely populated Nima (NM) and Jamestown (JT) (figure 6). In the current study (2019/2020), the mean annual PM_2.5_ concentrations were lower at the same locations, and ranged from 34 *μ*g m^−3^ at EL to 40 *μ*g m^−3^ in JT. This suggests a reduction (and more uniformity/plateau) in PM pollution in the city. The largest reductions were observed in high-density residential neighborhoods of JT and NM, where PM_2.5_ levels decreased on average by ∼60%. We observed a smaller reduction (35%) in the middle-income AD, but slight increase (21%) in high-income EL where there are a mix of residences and corporate, commercial and small businesses. The observed increase in high-income EL could also come from an overall increase in local commercial activities.

## 6. Discussion

We conducted a large-scale measurement campaign, and a detailed analysis of the spatial and temporal patterns of ambient PM_2.5_ and BC pollution in the SSA city of Accra (1500 km^2^). We found a reduction in PM_2.5_ pollution when compared with a decade ago, but the present levels exceed local and international public health guidelines by ∼2–4 folds. Our data show that PM_2.5_ pollution in Accra is becoming more uniform across communities, similar to cities in Europe and North America where PM_2.5_ is a regional pollutant and not as affected by community sources as in the past. Nonetheless, there remain some disparities in PM_2.5_ and BC concentrations within the city with significant seasonal variations. The CBI (mostly influenced by traffic) and high-density residential (mostly influenced by traffic and biomass use) areas were 35%–50% more polluted relative to peri-urban sites, which typically experience relatively lower traffic, commercial and industrial activities. Within-year changes in local meteorology produced distinct seasonality in PM_2.5_ and BC pollution, with concentrations during the Harmattan about twice that of the non-Harmattan period. Diurnal concentrations of PM_2.5_ peaked at dawn and dusk at times that coincided with the morning/evening traffic rush and biomass use hours.

In this city-wide analysis, our findings are consistent with previous smaller studies conducted in the AMA that also reported higher PM_2.5_ and BC concentrations at locations with persistent road-traffic and in densely populated neighborhoods [17, 23]. Similar to our results, studies in other large SSA cities have reported higher PM_2.5_ concentrations in locations with high road-traffic volumes in the CBI areas of Nairobi, Kenya [46] and Kampala, Uganda [47]; as well as higher PM_2.5_ and BC concentrations at industrial and high-density residential sites in Ibadan, Nigeria [48]. Within the sub-region, mean annual PM_2.5_ concentrations in our study are higher than annual averages observed for equally sprawling cities like Ibadan, Nigeria (24–33 *μ*g m^−3^) [48]. In global comparisons, mean annual PM_2.5_ in the GAMA were substantially higher than those found in large cities of high-income countries such as New York, USA (5–11 *μ*g m^−3^) [49] and London, UK (5–15 *μ*g m^−3^) [50], but lower than annual averages in Asian cities such as Beijing, China (53–112 *μ*g m^−3^) [51] and Delhi, India (122–148 *μ*g m^−3^) [52]. Although we did not study the composition and relative contribution of different sources to PM_2.5_ pollution, the high BC levels observed at CBI and high-density areas suggest that vehicle emissions and biomass burning are important determinants of PM_2.5_ pollution in the GAMA. Our observed city-wide spatial patterns aligns with the work of Zhou *et al*, 2013, which documented major contributions from traffic, road dust, and biomass burning to PM_2.5_ and BC pollution in the Accra city core.

Elevated PM_2.5_ during the Harmattan season is expected across West Africa given the influence of transported mineral dust from Sahara desert [5, 17, 23, 48, 53–55]. However, the observed increase in BC concentrations, a product of incomplete combustion, in the Harmattan season also suggests that changes to local meteorological conditions during this period (e.g. high temperature, low wind-speed and absence of precipitation) may produce stagnant conditions that substantially amplify local anthropogenic emissions [56]. The daily PM_2.5_ cycle of bimodal pattern with peaks in the mornings and evenings, provides further support for the influence of rush hour traffic, biomass combustion as well as pollution build-up due to temperature inversion and variations in meteorological conditions between day and nighttime hours and seasons [23, 57]. It is likely that the observed improvements in PM_2.5_ pollution, especially in high-density neighborhoods that also tended to have high-biomass use, was due to gradual reductions in biomass use. Both behavioral and policy changes accompanying economic improvements might have brought about reduction in local community emissions. For instance, there is evidence of downward trend in the proportion of households utilizing biomass fuel for cooking, with a significant switch from predominantly wood (more polluting) to charcoal and gas, which are less polluting [58]. In terms of policy, Ghana currently has in place penalties on the importation of used and old vehicles to curb traffic emissions in general [20]. Therefore, incentivizing transition to cleaner fuels could further improve air quality in the GAMA [6, 59, 60]. With sustained economic and urban expansion, vehicle ownership in Ghana is increasing by 10% annually [61] and the GAMA accounts for 60% of the total number of registered vehicles [21, 22]. Without investments in infrastructure (e.g. improved road networks) and environmental management programs, this growth could lead to higher vehicular emissions than observed previously [17], which will worsen air quality over time. Attaining cleaner air in Accra (i.e. meeting WHO guideline levels) is likely to require implementation of Ghana’s proposed traffic-related air pollution reduction strategies such as the bus-rapid transit system, development of vehicle emission standards, and maintaining the current penalties on importation of old vehicles while providing incentives and rebates on new cars [21, 22]. Given that PM_2.5_ and BC pollution are worse during Harmattan, there is a specific need for additional air quality management plans in this period. Land stabilization interventions (such as covering road surfaces with dust suppressants, and sweeping/ washing roads) and building extensive green walls of forest to act as protective barrier around the city can reduce dust particle load [62].

## 7. Conclusion

As urbanization in SSA continues and cities are faced with the challenge of managing air quality from diverse sources [8], data on local air pollution and sources are urgently needed to enable evidence-based policy efforts to protect public health. To avoid similar poor air quality challenges seen in Asian cities, systematic air quality management plans are needed to further reduce current air pollution levels. Successful air pollution mitigation efforts will require attention to land-use planning and accounting for seasonality. Besides the direct impact of Harmattan on PM pollution, changes in the local meteorology during this period suggests almost no room for worsening emissions from local sources during this period. Our study provides compelling evidence for systematic air pollution monitoring as well as implementation of Ghana’s air quality policy initiatives aimed at protecting health and improving air quality in the GAMA.

## Data Availability

The data that support the findings of this study are available upon reasonable request from the authors.
